# F207. SCHIZOTYPY AND SENSORY GATING: A 6-MONTH-OLD EEG STUDY

**DOI:** 10.1093/schbul/sby017.738

**Published:** 2018-04-01

**Authors:** Eleanor Smith, Trevor Crawford, Megan Thomas, Vincent Reid

**Affiliations:** 1 Lancaster University; 2 Blackpool Victoria Teaching Hospital NHS Foundation Trust

## Abstract

**Background:**

Schizotypal traits are present in the general population and are distributed along a continuum, with the clinical disorder schizophrenia found at its extremity (Claridge, 1997). Schizotypy is a dimension of personality within the general population, which has been found to be elevated among schizophrenia-spectrum patients (Brosey and Woodward, 2015) and their first-degree relatives (Morenzo-Izco et al., 2015). One hypothesis to account for the sensory deficits observed across the spectrum suggests a difficulty in the inhibition of irrelevant sensory input, such as the secondary beep in the paired-click paradigm.

Sensory gating describes the pre-attentional habituation of responses to repeated sensory input, for example, auditory tones. This gating mechanism is used to distinguish between important and irrelevant information (Hall et al., 2011) and is typically explored using the paired-click paradigm and analysed using the P50 event-related potential component. This can be observed approximately 50-milliseconds following the presentation of an auditory stimulus and is a highly established biological trait of schizophrenia, with abnormalities displayed in the P50 component all throughout the schizophrenia-spectrum.

**Methods:**

This research aimed to observe whether the 6-month-old offspring of mothers with schizotypic traits display abnormalities in the P50 event-related component when explored using the paired-click paradigm. The paired-click paradigm was used to highlight the sensory-gating abilities of fifty-three 6-month-old infants during 15-minutes of continuous sleep. The mothers of the infants completed the Short Form of the Oxford and Liverpool Inventory of Feelings and Experiences, which was used to determine their personality dimension scores, and identify schizotypic traits. Participants were categorized into one of three groups: infants of controls mothers, infants of intermediate mothers, and infants of schizotypic mothers.

**Results:**

It was predicted that the 6-month-old infants of mothers who demonstrate schizotypy scores would demonstrate different amplitudes compared to those of control mothers. This research found a significant generalized difference between the P50 component for the paired-clicks in the right hemisphere of the brain (F(1,51)=5.34, p=.025), and a significant latency effect was observed in the frontal regions (F(1,51)=5.41, p=.024). A significant between-subjects effect was observed centrally (F(2,50)=3.71, p=.031); suggesting there are significant differences between the ways each group distinguished the paired-clicks. Infants of schizotypic mothers showed an increase in activation compared to other groups. An interaction was observed in the left hemisphere between the paired-clicks and each identifiable group (F(2,50) = 3.45, p = .039). In addition to the P50 a significant slow wave effect was also observed across the left (F(1,51)=8.38, p=.006) and right (F(1,51)=7.81, p=.007) posterior regions; a latency effect in the left (F(1,51)=5.47, p=.023), and a distinction in mean amplitude in the right (F(1,51)=7.25, p=.010).

**Discussion:**

Schizotypy is viewed as a risk factor for schizophrenia, which is present in the general population, and is present on the schizophrenia-spectrum. The 6-month-old infants of mothers showed an increase in activity centrally, demonstrating that the infants’ P50 amplitudes were influenced by their mothers’ schizotypy status. This finding is consistent with the developmental hypothesis of schizophrenia, however longitudinal studies will be required to determine whether a sensory gating deficit is a valid predictor of the later onset of schizophrenia.

